# Immune Resolution Dilemma: Host Antimicrobial Factor S100A8/A9 Modulates Inflammatory Collateral Tissue Damage During Disseminated Fungal Peritonitis

**DOI:** 10.3389/fimmu.2021.553911

**Published:** 2021-02-26

**Authors:** Madhu Shankar, Nathalie Uwamahoro, Emelie Backman, Sandra Holmberg, Maria Joanna Niemiec, Johannes Roth, Thomas Vogl, Constantin F. Urban

**Affiliations:** ^1^ Department of Clinical Microbiology, Umeå Centre for Microbial Research (UCMR), Umeå University, Umeå, Sweden; ^2^ Molecular Infection Medicine Sweden (MIMS), Umeå University, Umeå, Sweden; ^3^ Department of Medical Chemistry and Biophysics, Umeå University, Umeå, Sweden; ^4^ Institute of Immunology, Universitätsklinikum Münster, University of Münster, Münster, Germany

**Keywords:** sepsis, host-pathogen interactions, peritonitis, *Candida albicans*, inflammation, S100A8/A9 complex, host-targeted agents

## Abstract

Intra-abdominal infection (peritonitis) is a leading cause of severe disease in surgical intensive care units, as over 70% of patients diagnosed with peritonitis develop septic shock. A critical role of the immune system is to return to homeostasis after combating infection. S100A8/A9 (calprotectin) is an antimicrobial and pro-inflammatory protein complex used as a biomarker for diagnosis of numerous inflammatory disorders. Here we describe the role of S100A8/A9 in inflammatory collateral tissue damage (ICTD). Using a mouse model of disseminated intra-abdominal candidiasis (IAC) in wild-type and S100A8/A9-deficient mice in the presence or absence of S100A9 inhibitor paquinimod, the role of S100A8/A9 during ICTD and fungal clearance were investigated. S100A8/A9-deficient mice developed less ICTD than wild-type mice. Restoration of S100A8/A9 in knockout mice by injection of recombinant protein resulted in increased ICTD and fungal clearance comparable to wild-type levels. Treatment with paquinimod abolished ICTD and S100A9-deficient mice showed increased survival compared to wild-type littermates. The data indicates that S100A8/A9 controls ICTD levels and antimicrobial activity during IAC and that targeting of S100A8/A9 could serve as promising adjunct therapy against this challenging disease.

## Introduction

In surgical patients, peritonitis frequently results in severe sepsis, particularly in the intensive care unit (1), where more than 70% of patients suffering from this complication may succumb to death within 72 h (2). Hence, treatment options are required that extend life expectancy and allow proper treatment (3). Peritonitis is characterized by an inflamed membrane of the abdominal cavity, the peritoneum, and is caused by endogenous microbes of the gastrointestinal microbiota (4). Intra-abdominal surgery frequently originates peritonitis (5). The surgical disruption of mucosal barriers facilitates microbial translocation from the gut lumen into the peritoneal cavity and from there into circulation (1, 6). The microbial spread leads to deep-seated infections by gut-colonizing organisms, such as the yeast *Candida albicans* (7). Notably, intra-abdominal candidiasis (IAC) is the most common non-mucosal fungal disease among hospitalized patients and is associated with fungal cells disseminating to the liver and other organs *via* lymphatics or bloodstream (8). Accordingly, liver tissue damage and leukocytosis are hallmarks of deep-seated and systemic *C. albicans* infections (9). As of today, IAC remains challenging to diagnose and thus results in high mortality rates from 25% to 60% (5).

Upon microbial translocation, the human body prompts an inflammatory response with the aim to remove invading microbes from the peritoneal cavity and circulation. On a molecular level, the recognition of pathogenic microbes activates a host immune response that induces the release of pro-inflammatory cytokines and chemokines including interleukin-6 (IL-6), macrophage inflammatory protein 1α (MIP-1α or CCL-3), and tumor necrosis factor (TNF) (10). These cytokines recruit phagocytes to the peritoneum, where they attempt to engulf and kill the microbes. To further restrict the infection, abscess development is promoted *via* the production of fibrinous exudate.

The innate immune system’s inflammatory response is a double-edged sword that has beneficial and detrimental outcomes, such as pathogen clearance or inflammatory collateral tissue damage (ICTD), respectively (11). Failure to locally confine the causative agents of peritonitis, as well as associated ICTD, may lead to organ failure, coma, and death (8). However, most infection studies have neglected the possibility to target ICTD as a complementary approach. Such a host-targeted therapy may enhance tolerance during inflammation, restore immune homeostasis, and thereby enable the patient to overcome the infection (12).

The resolution of infection is an active process involving reprogramming of cells and modulation of immune mediators (13), such as S100A8/A9. This EF-hand calcium-binding heterodimer protein complex, also known as calprotectin, is an antimicrobial factor released by activated, stressed, or necrotic cells. Physiologically, S100A8/A9 has multiple functions ranging from pro- and anti-inflammatory to antimicrobial activities (14).

As pro-inflammatory immune mediator, S100A8/A9 acts as alarmin or damage-associated molecular pattern (DAMP) that binds pattern recognition receptors (PRRs), such as Toll-like receptor-4 (TLR4) and consequently induces expression of pro-inflammatory cytokines and chemokines (15–17). The q-compound paquinimod (Paq), an immunomodulatory molecule designed to treat chronic inflammatory diseases, prevents the binding of S100A9 to TLR4 and thus abrogates the pro-inflammatory activity of S100A8/A9 (18–23). In contrast to the aforementioned pro-inflammatory mode, a prolonged presence of S100A8/A9 leads to a state of immune tolerance as counter mechanism (16). Under calcium-rich conditions in extracellular milieus, S100A8/A9 heterodimers form tetramers, which are unable to trigger pro-inflammatory responses. This anti-inflammatory mechanism serves as natural safeguard to restrict S100A8/A9 activity and to prevent overwhelming immune reactions (17). To avoid quick inactivation of S100A8/A9 under experimental, usually calcium-rich conditions, S100A8 homodimers, which do not form tetramers, are widely used to mimic heterodimer activity (17).

Furthermore, S100A8/A9, functions as antimicrobial factor. Deployed by leukocytes, S100A8/A9 firmly binds micronutrients including zinc and manganese ions to deprive microbes of these essential nutrients (24, 25). Neutrophils release the heterodimer during the formation of neutrophil extracellular traps (NETs) to capture and eradicate *C. albicans* (26). Notably, S100A8/A9 has distinct binding sites for Ca^2+^ on the one hand and for Zn^2+^ and Mn^2+^ on the other hand. Ca^2+^ binding is important for the regulation of proinflammatory activity, whereas Zn^2+^ and Mn^2+^ scavenging mediates the antimicrobial activity of the protein complex (27). Hence, pharmacological inhibition of S100A8/A9 binding to PRRs does not necessarily prevent the antimicrobial activity mediated by Zn^2+^ and Mn^2+^ binding (14).

In addition to its physiological functions, S100A8/A9 can be utilized as inflammation marker in clinical diagnostics. As the most abundant cytoplasmic protein of human neutrophils it is released upon cell activation. Therefore, quantification of S100A8/A9 is frequently used to monitor neutrophil elevation in various inflammatory diseases (28).

In the present study, we used a mouse model of *C. albicans* peritonitis in order to characterize the role of S100A8/A9 during inflammation and elimination of fungal burden. By comparing S100A8/A9-deficient and wild-type (WT) mice, we show that the local inflammatory response failed to contain the pathogen in the peritoneum and led to detrimental ICTD in the liver dependent on the presence of S100A8/A9. In order to interfere with anti-inflammatory properties of S100A8/A9, mice were treated with Paq, which abrogated S100A8/A9-induced ICTD and yet had no negative impact on fungal clearance. This suggests that Paq may be used as adjunct therapy option during severe IAC.

## Materials and Methods

### Statistical Analyses

Statistical analysis was conducted using GraphPad Prism 6 software. For comparison of the TNF ELISA with primary immune cells, an unpaired, two-tailed student’s *t*-test was used. For multiple comparisons one-way ANOVA was used. Tukey’s multiple comparison test was applied, when all groups were to be compared. Sidak’s multiple comparison test was applied, when specific groups were to be compared. Dunnett’s multiple comparison test was applied, when all groups were compared to one control group. A two-way ANOVA with Tukey’s multiple comparison test was used to analyze the different plasma parameters as measured by Vetscan VS2. *In vivo* experiments comparing levels of plasma alanine aminotransferase (ALT) across groups and colony-forming units (CFUs) including WT and S100A8/A9-deficient (*S100A9* ^-/-^) mice were analyzed using the non-parametric, unpaired Mann-Whitney test. Mouse survival was analyzed using the log-rank test. In all comparisons, the sample size is specified in figure legends, and a p<0.05 was considered significant with * equaling p < 0.05, **p < 0.01, ***p < 0.001, ****p < 0.0001.

### Yeast Strains and Growth Conditions

*C. albicans* clinical isolate strain SC5314 was cultured overnight in YEPD (1% yeast extract, 2% bacto-peptone and 2% glucose) at 30°C. *C. albicans* cells were washed three times in PBS prior use in all assays. Cell numbers were calculated using Vi-CELL Cell Viability Analyzer (Beckman Coulter AB).

### Animal Infections

All mice were maintained according to a previous report (29) at Umeå Centre for Comparative Biology (UCCB), Umeå University, Umeå, Sweden. Inbred C57BL/6 mice served as WT strain and inbred *S100A9^-/^* ^-^ strain, which was obtained from University of Münster, with the same genetic background as C57BL/6 served as S100A8/A9-deficient strain. Although S100A8-coding RNA is expressed in these mice, the protein is quickly degraded, as the partner protein S100A9 is missing (30). The mice are, therefore, completely deficient of the heteroduplex. If not stated otherwise, mice were infected intraperitoneally (IP) with 3 × 10^6^ C*. albicans* cells per g mouse from an overnight culture in YEPD. For Paq treatment, mice received the compound solved in PBS by IP injection shortly after infection for a final concentration of 30 mg/kg mouse. In survival experiments, injection was repeated every 24 h. To revert lack of S100A8/A9 recombinant S100A8 protein (rS100A8) was administered by IP injection into *S100A9^-/^* ^-^ mice shortly after infection for a final concentration of 5 µg protein/g mouse. For intravenous infection (IV), mice were challenged intravenously with 2.5 × 10^4^ C*. albicans* cells per g mouse.

### Isolation and Differentiation of Bone Marrow-Derived Macrophages

Bone marrow-derived macrophages (BMDMs) were differentiated from mouse bone marrow cells as described in a previous report (31). Viability of Paq-treated BMDMs was analyzed with propidium iodide by flow cell cytometry (FACS) using a BD LSR II flow cytometer (BD Biosciences, San Jose, CA) (31). All Paq concentrations used are indicated in the figure legends of the respective experiments. A concentration of 10 µg/ml rS100A8 was used to complement *S100A9^-/^* ^-^ BMDMs. Prior to each experiment, the BMDM viability was routinely monitored and remained above 90% for both mouse genotypes.

### Fungal Burden in Organs of Infected Mice

**Figure 1:**
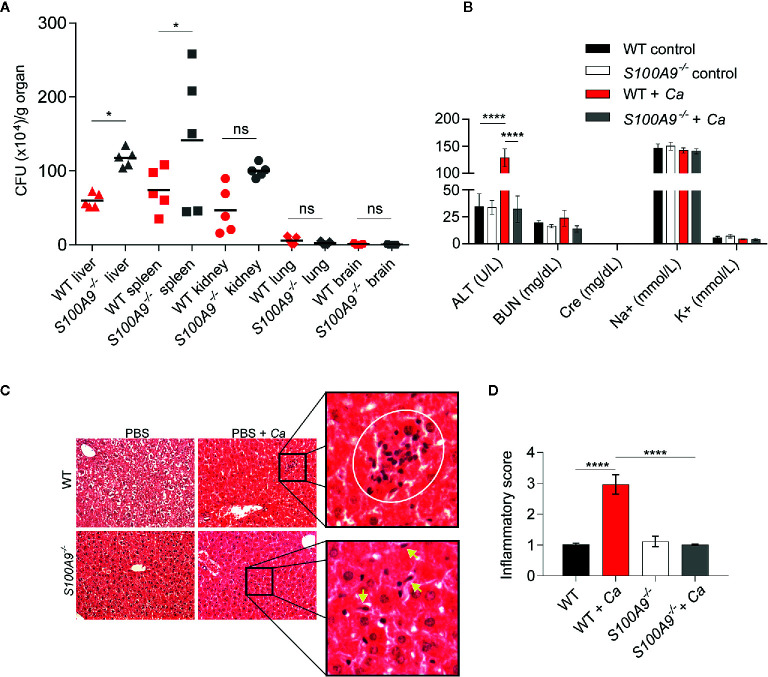
Disseminated *C. albicans* peritonitis mouse model depicts the importance of S100A8/A9 in fungal dissemination and ICTD in the liver. Intraperitoneal *C. albicans* infection (3 × 10^6^ cells per g mouse) in WT or S100A8/A9-deficient (*S100A9* ^-/-^) mice is shown after 24 h. As described in the text, the knockout mouse strain completely lacks protein expression of both subunits of the S100A8/A9 complex. **(A)** *C. albicans* dissemination into liver, spleen, kidney, lung and brain was determined by CFUs per g organ. Fungal burden is higher in liver and spleen from *S100A9* ^-/-^ than from WT mice. Data is from one of two experiments using n = 5 mice per group and shown as means. Statistical significance was evaluated using a one-way ANOVA with Sidak’s multiple comparison test **(B)** To determine organ damage, plasma was harvested from infected animals and uninfected controls. Plasma (100 µl) was analyzed using a Vetscan VS2 clinical chemistry system. Shown are values for alanine aminotransferase (ALT), blood nitrogen urea (BUN), creatinine (Cre), sodium (Na+), and potassium (K+). Only ALT, indicative of liver damage, was significantly increased in infected WT and but not in *S100A9* ^-/-^ mice. Data are from one of four experiments using n = 6 mice for infected and n = 3 mice for uninfected mice and presented as means while error bars depict standard deviation (SD). Statistical significance was evaluated using a two-way ANOVA with Tukey’s multiple comparison test. **(C)** Analysis of histological liver tissue sections from infected animals revealed that lack of S100A8/A9 led to reduced inflammation and leukocyte infiltration zones in the organ. Shown are representative photomicrographs of hematoxylin-eosin (H&E) stained liver sections of uninfected and infected WT and *S100A9* ^-/-^ mice. Zoomed images show increased inflammatory leukocyte infiltrates (white circle) in WT-infected cells (top panel). Yellow arrows indicate fungal cells (bottom panel) at 20× magnification. CV: central vein, PV: portal vein. **(D)** Quantification of **(C)**. An inflammatory score of H & E-stained liver sections of uninfected and infected WT and *S100A9^-/-^* mice. A score of 2 = moderate cell infiltration, >3 = large number of infiltrates, 4 = full tissue inflammatory infiltration. Data is from 10× microscopy image fields of two sections from mice n = 2 (WT and *S100A9* ^-/-^) and n = 6 (WT + *Ca* and *S100A9* ^-/-^ *+ Ca*) from two independent experiments. Error bars indicate SD as evaluated using a one-way ANOVA with Tukey’s multiple comparison test, statistical significance with *equals p-value < 0.05, ****p-value < 0.0001, and ns, not significant.

To determine the fungal burden in infected organs, analyses were conducted according to a previous report (32) Briefly, IP-infected mice were sacrificed after 24 h and livers and as implied for the experiments in **Figure 1** additionally spleens, kidneys, lungs, and brains were removed, weighted and subsequently homogenized using a gentleMACS™ dissociator (run 12, two times per organ). Organ homogenates were kept on ice. Serial dilutions of homogenates (100 µl) were plated on YEPD plates, and the number of CFUs per g organ was assessed to determine the fungal burden.

### Histological Preparations

Histological preparations and inflammatory score analyses were conducted as in previous reports (26, 33). To determine the inflammatory score, whole hematoxylin-eosin (H & E) stained sections were analyzed for inflammation and scored under the supervision of a specialized animal pathologist. The sections from each animal were evaluated and received score = 0, when no inflammatory cells were present in the tissue, score = 1, for a few inflammatory cells (1–20 cells), score = 2, for moderate cell infiltration (21–40 cells), score = 3, for a large number of inflammatory cells (41–60 cells), and score = 4, when inflammation was spread entirely over the tissue (61 cells) (33).

### Clinical Chemistry Analysis in Plasma Samples

To assess organ damage, blood from mice was collected *via* cardiac puncture and plasma was separated by centrifugation. Mice received 200 µl heparin diluted in PBS to 1000 international enzyme units/ml subcutaneously 15 min before blood drawl (LEO Pharma Nordic, Malmö, Sweden). Mice were anesthetized by IP injection of Dormitor (1 mg/kg) and Ketalar (75 mg/kg) in 150 µl PBS per mouse. After the mice lost their reflexes, blood was harvested by cardiac puncture using a 26G Sub-Q syringe (Becton Dickinson, Stockholm, Sweden). The retrieved blood was stored in heparinized, 2-ml tubes at room temperature. Blood nitrogen urea (BUN), creatinine (Cre), sodium (Na^+^), potassium (K^+^), and alanine aminotransferase (ALT) levels were measured using a Vetscan VS2™ system (SCIL animal care company) as previously reported (32).

### Cytokine and Chemokine Quantification

BMDMs were seeded at 1 × 10^5^ cells per well in 96-well microplates in RPMI cell culture medium and then infected with *C. albicans* at MOI 1 for 24 h. Cell-free culture supernatants were harvested and analyzed for indicated cytokines or chemokines by ELISA (BioLegend-ELISA MAX™, San Diego, CA, USA), or by Pro-Mouse Cytokine Bio-Plex™ customized assay (Bio-Rad Laboratories, Solna, Sweden) according to the manufacturer’s instructions. Values from raw data were used in the respective standard curve equations to calculate the according cytokine concentrations. In some instances, negative values could arise, when concentrations were calculated from low raw data values very close to media background. Hence, negative concentration values should be regarded as concentrations below detection limit.

For determination of cytokine and chemokine levels in livers of mice, livers from IP-infected and uninfected control animals were harvested, washed once in cold HBSS and homogenized in fresh cold HBSS using a gentleMACS™ dissociator (Miltenyi Biotec, Bergisch Gladbach, Germany). Suspensions were frozen at -80°C, thawed and centrifuged at 3000 × g for 15 min. Supernatants were collected and kept on ice until analysis. Prior to quantification, the samples were diluted 1:2 and subjected to a Pro-Mouse Cytokine Bio-Plex™ 23-Plex assay (Bio-Rad Laboratories, Solna, Sweden) according to the manufacturer’s instructions. Average values from uninfected control liver homogenates were subtracted from infected liver homogenates to account for different background values unrelated to *C. albicans* infection.

### Myeloperoxidase Activity Assay

To quantify neutrophil (granulocyte) infiltration livers were harvested, washed in cold HBSS and homogenized in fresh cold HBSS using a gentleMACS™ dissociator (Miltenyi Biotec, Bergisch Gladbach, Germany). The homogenates were frozen at -80°C, thawed and centrifuged at 3000 × g for 15 min. Myeloperoxidase (MPO) activity was assessed from the supernatants with 3,3′,5,5′-tetramethylbenzidine (TMB from Sigma Aldrich, St Louis, MO, USA) (34). Briefly, 10 µl sample were combined with 80 µl 0.75 mM H_2_O_2_ and 110 µl TMB solution (2.9 mM TMB in 14.5% DMSO and 150 mM sodium phosphate buffer at pH 5.4), and the plate was incubated at 37°C for 5 min. The reaction was stopped by adding 50 µl 2 M H_2_SO_4_ (Sigma), and absorption was measured at 450 nm to estimate MPO activity. To obtain values per organ the total volume of each liver suspension was measured. Values of livers from uninfected control animals were subtracted as background prior to plotting of the data.

### Generation of Recombinant S100A8

The *S100A8* and *S100A9* gene encoding for monomers of the mouse dimer S100A8/A9 (UniProt P27005, P31725) were synthesized, codon-harmonized and purchased from DNA2.0. The genes were GST-tagged and cloned into *Escherichia coli* BL21 strain. The cloned cells were auto-induced overnight. The cells were pelleted and suspended in 10 mg/ml of 1×PBS supplemented with DNase and protease inhibitors. The cells were lysed on ice by sonication (Branson Digital sonifier; 10 mm horn, 50% power) for 6 min with alternating 10 s pulses and pauses. The lysate was clarified by centrifugation at 23,000 × g for 20 min at 4°C. The clarified lysate was filtered through a 0.45 µm syringe filter and batch bound to 2.0 ml GST Sepharose (~25 mg protein/ml) for 2 h at 4°C gravity flow over a column and washed with 30 column volumes of PBS. Samples were eluted in 4–6 ml fractions with 50 mM Tris pH 8 and 10 mM glutathione. For each fraction, the resin was incubated with elution buffer for 10 min prior to collecting the flow through. The fractions were analyzed at A_280_. The pooled fractions of protein were cleaved (1:100) using protease 3c (PreScission^®^Protease) to remove the GST tag, and the fractions of GST tag-free protein were pooled. The protein was then separated by size-exclusion chromatography (SEC) using a Superdex 75 16/600 column (GE Healthcare Life Sciences, UK) equilibrated with PBS pH 7, at a flow rate of 0.5 ml/min. The fractions containing purified S100A8 (rA8) and S100A9 (rA9) were concentrated using an Amicon Ultra-4 centrifugal filter device with a 3 kDa molecular-weight cutoff (Merck, Darmstadt, Germany). The primary sequence, the intact mass and the presence of product were confirmed by mass, spectrometry using an ABI 4800 MALDI tandem time-of-flight mass spectrometer (Applied Biosystems, Waltham, MA, USA). Recombinant proteins were screened for endotoxin contamination, and levels were below 0.6 pg per µg protein, as previously recommended (35).

## Results

### A Disseminated Fungal Peritonitis Model to Determine Immune-Modulatory Roles of S100A8/A9

We used a murine model of IAC to study the immunological contributions of S100A8/A9 during a clinically-relevant form of candidiasis. For this, WT and *S100A9^-/-^* mice were IP-infected with *C. albicans* and fungal dissemination into different organs was determined 24 h post infection (**Figure 1A**). Liver, spleen, and kidney showed considerable fungal burden (approximately 5 × 10^5^ CFUs per g organ) with a significantly higher level of dissemination in livers and spleens from *S100A9* ^-/-^ than from WT mice. Fungal dissemination to lungs and brains was negligible (**Figure 1A**). To assess possible organ damage caused by experimental IAC, we analyzed plasma from IP-infected mice 24 h post infection for clinical disease markers using a Vetscan VS2 device (**Figure 1B**). We determined ALT, indicative for liver injury, BUN and Cre, both indicative for kidney injury, as well as Na^+^ and K^+^ ion concentration, both indicative for dehydration and renal dysfunction (**Figure 1B**). Interestingly, only ALT levels were significantly increased in IP-infected WT (>130 U/L), but not in infected *S100A9* ^-/-^ mice (<40 U/L), in which the values remained at basal levels. Indicators for kidney injury and renal dysfunction where not elevated above basal levels in both infected genotypes (**Figure 1B**). Therefore, we focused our investigations on the role of S100A8/A9 on liver ICTD during experimental IAC. We additionally noted that IP-infected WT mice frequently developed ocular discharge (“pus eye”), which was significantly reduced in IP-infected *S100A9* ^-/-^ mice (**Figure S1A**). This is remarkable, since fungal burden in liver and spleen was higher in these animals, whereas dissemination to lung and brain remained low in both genotypes (**Figure 1A**).

To corroborate findings on liver damage during experimental IAC, we performed histological examination of liver tissue. For this purpose, we IP-infected WT and *S100A9* ^-/-^ mice with *C. albicans*, collected livers 24 h post infection and prepared H & E stained liver sections. Microscopically, WT liver sections indicated higher numbers of leukocyte infiltration zones compared to *S100A9* ^-/-^ sections (**Figure 1C**, top panel and **Figure S1B**). More histological tissue sections were analyzed to obtain an inflammatory score (**Figure 1D**) which showed increased inflammatory cell infiltrates (level 3) in *C. albicans*-infected WT samples compared to a virtual lack of inflammation (level 1) observed in samples from *S100A9* ^-/-^ mice (see **Figure S1B** for reiteration of quantification presented in **Figure 1D**). In addition, granulocyte infiltration was quantified *via* MPO activity in liver homogenates. MPO activity was also reduced in liver homogenates from *S100A9* ^-/-^ compared to their WT counterparts (**Figure S2A**). Yet, this difference was not statistically significant, suggesting that not only the numbers of leukocyte, but also their local accumulation and possibly activation are important for the assessment. Nevertheless, there was visual evidence of increased fungal burden in the *S100A9^-/-^* livers compared to WT (**Figure 1C**, bottom panel and **Figure S1B**) confirming the antimicrobial role of S100A8/A9.

In additional, independent experiments we IP-infected WT and *S100A9^-/-^* mice with *C. albicans* and assessed plasma ALT levels and liver CFUs (**Figures S2B, C**). As mentioned, ALT levels from plasma samples are an indicator of liver damage (1, 36), whereas the host’s ability to clear infections correlates to amounts of CFUs (37). The results corroborated that 24 h post infection liver damage was consistently higher in IP-infected WT mice (**Figure S2B**), even though fungal burden was significantly lower in livers of WT mice than in those of *S100A9^-/-^* mice (**Figure S2C**). Consequently, we defined ICTD during experimental IAC from here on as the significant elevation of plasma ALT levels with frequent occurrence of ocular discharges as an indication of systemic inflammation. In addition, lethargy could be observed in WT but not in *S100A9^-/-^* mice (**Video S1**). This demonstrates that experimental IAC in WT mice rapidly leads to acute, severe disseminated infection reflecting clinical manifestations characteristic of IAC in humans (9).

### Paq Reduces and rS100A8 Increases Cytokine Release of *C. albicans*-Infected Macrophages

**Figure 2:**
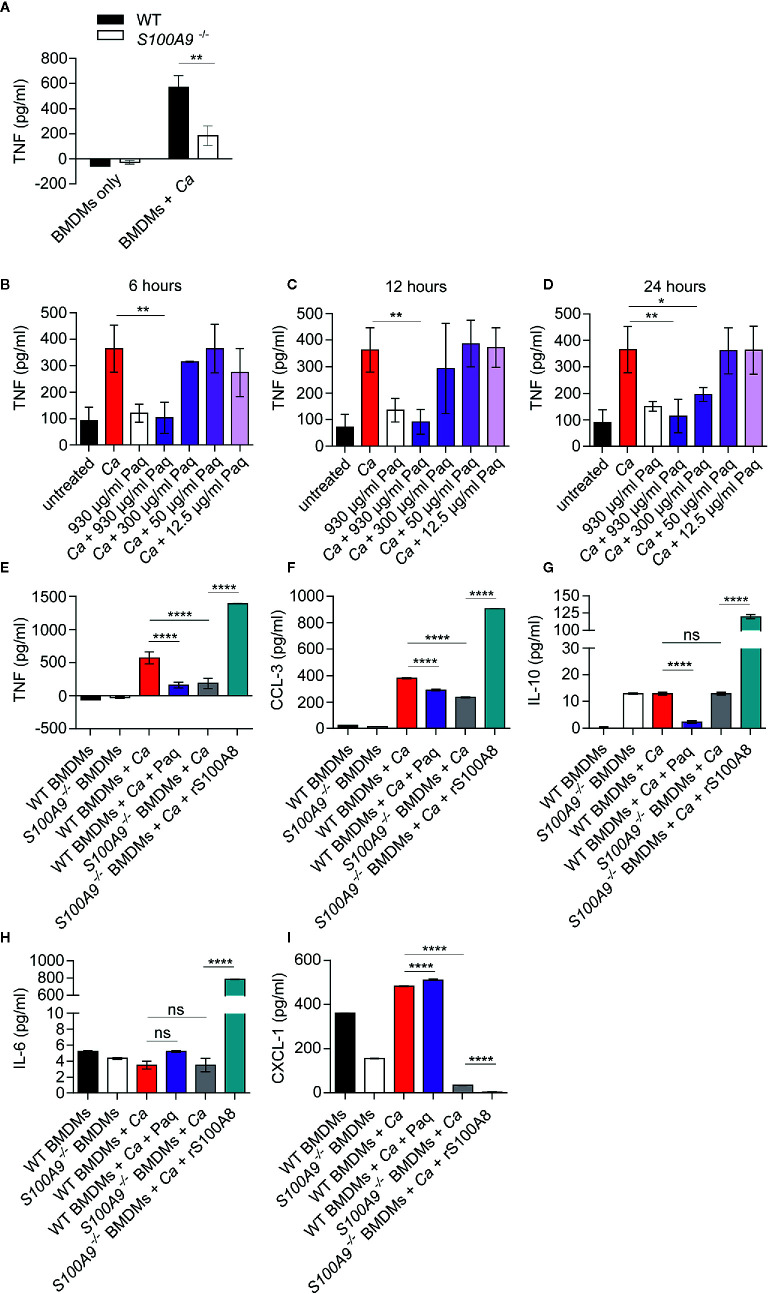
Lack of S100A8/A9 and addition of Paq reduce pro-inflammatory cytokine release by WT BMDMs, whereas supplementation with rS100A8 restores cytokine release by *S100A9* ^-/-^ BMDMs upon *in vitro C. albicans* infection. **(A)** Mouse BMDMs of WT and *S100A9^-/-^* from different donors were incubated with *C. albicans* (*Ca*) at MOI 2. After 24 h cytokines were measured in supernatants. Shown are mean levels of TNF and error bars indicate SD from three biological replicates with technical triplicates from one of two experiments. Statistical significance was analyzed using an unpaired two-tailed *t*-test with Welch’s correction. **(B–D)** WT BMDMs from three different mice were incubated with *C. albicans* (MOI 2), with different Paq concentrations (930–12.5 µg/ml) and after 6, 12, and 24 h TNF was measured in the supernatant by ELISA. Shown are means ± SD from three independent experiments with technical triplicates. Statistical differences were analyzed using a one-way ANOVA with Dunnett’s multiple comparison test. **(E–I)** WT and *S100A9* ^-/-^ BMDMs were infected with *C. albicans* (MOI 1) and treated with 930 µg/ml of Paq or left untreated (WT BMDMs + *Ca* ± Paq) and supernatants were collected 24 h post infection. Similarly, *S100A9* ^-/-^ BMDMs were infected with *C. albicans* (MOI 1) and treated with rS100A8 or left untreated (*S100A9* ^-/–^ BMDMs + *Ca* ± rS100A8) and supernatants were collected 24 h post infection. Cytokine and chemokine concentrations in these supernatants were determined using a Pro-Mouse Cytokine Bio-Plex™ customized assay. Shown are means ± SD from four biological replicates with technical duplicates. Statistical significance was analyzed using a one-way ANOVA with Tukey’s multiple comparison test, statistical significance with * equals p-value < 0.05, **p-value < 0.01, ****p-value < 0.0001, and ns, not significant.

Macrophages are immune sentinels for early recognition of infection and upon pathogen contact they trigger signaling *via* the release of pro-inflammatory cytokines, such as TNF. Uncontrolled macrophage activation, however, may cause collateral damage (38). To determine how S100A8/A9 contributes to the TNF response of macrophages, we infected BMDMs from WT and *S100A9* ^-/-^ mice with *C. albicans in vitro* (**Figure 2A**). Higher levels of TNF (3.1 fold of average levels measured) upon *C. albicans* infection were induced by WT BMDMs (570 pg/ml) compared to *S100A9* ^-/-^ BMDMs (185 pg/ml), suggesting a reduced TNF response of infected macrophages lacking S100A8/A9. Since TNF is a pro-inflammatory cytokine, decreased TNF concentrations could possibly contribute to the reduced ICTD severity observed in IP-infected *S100A9* ^-/-^ mice (**Figure 1D**).

We thus aimed to assess whether the anti-inflammatory compound Paq could also block the *C. albicans*-induced TNF response of BMDMs. Paq specifically targets the subunit S100A9 and thereby prevents pro-inflammatory activity of the heteroduplex (39). At first, we determined the concentration at which Paq could reduce TNF release from *C. albicans*-infected BMDMs to basal levels after 6, 12, and 24 h (**Figures 2B–D**). Consistently, at a concentration of 930 µg/ml, there was significant TNF reduction compared to untreated infected BMDMs (**Figures 2B–D**). Although, after 24 h, 300 and 930 µg/ml Paq concentrations showed significant reduction of TNF release compared to untreated samples (**Figure 2D**), we decided to use 930 µg/ml for subsequent experiments. Notably, in the concentration range used, we did not observe any toxic effect of Paq on BMDMs (**Figure S3**).

**Figure 3:**
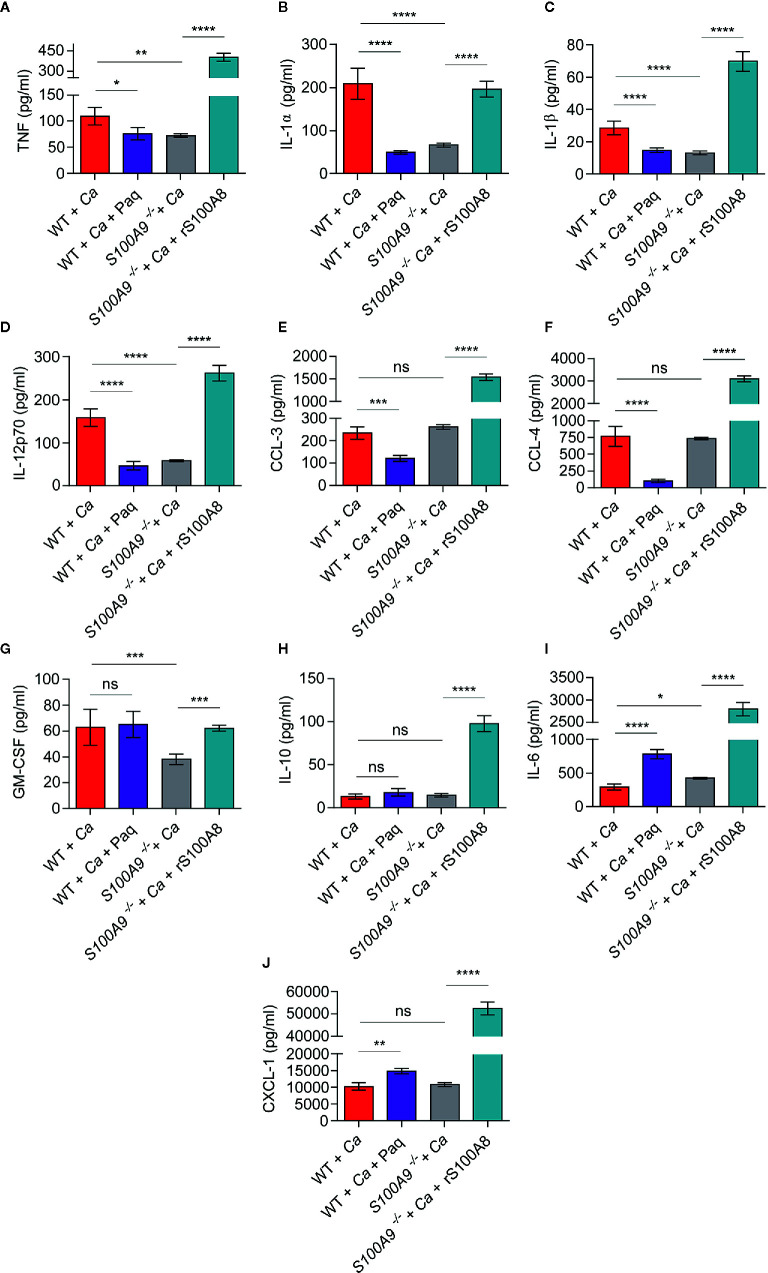
*In vivo* liver tissue cytokine levels from infected WT mice treated or untreated with Paq and *S100A9* ^-/-^ mice treated or untreated with rS100A8 unravel a role of S100A8/A9 for pro-inflammatory cytokine regulation during experimental IAC. To corroborate the effect of S100A8/A9 and Paq treatment on cytokine production possibly related to ICTD we IP-infected mice with 3 × 10^6^ C*. albicans* per g mouse, harvested the livers and determined cytokines and chemokines using a Pro-Mouse Cytokine Bio-Plex™ 23-Plex assay. Data is shown from n = 6 mice per group as means ± SD from two different experiments. To account for background correction, cytokine concentrations from livers of n = 2 uninfected mice per group, matching the treatments, were directly subtracted from the values of the respective infected animals. Statistical significance was evaluated using a one-way ANOVA with Sidak’s multiple comparison test. Different cytokines and chemokines are shown in individual graphs with **(A)** TNF, **(B)** IL-1α, **(C)** IL-1β, **(D)** IL-12, **(E)** CCL-3, **(F)** CCL-4, **(G)** GM-CSF, **(H)** IL-10, **(I)** IL-6, **(J)** CXCL-1. TNF, IL-1 α, IL-1β, and IL-12 are decreased in *S100A9* ^-/-^ and Paq-treated WT mouse livers, whereas they are increased in untreated WT and rS100A8-treated *S100A9* ^-/-^ mouse livers, statistical significance with * equals p-value < 0.05, **p-value < 0.01, ***p-value < 0.001, ****p-value < 0.0001, and ns, significant.

To assess how Paq influences other cytokines and chemokines released by macrophages upon *C. albicans* infection, we included analyses of cytokines typically released during early inflammatory responses. We chose anti-inflammatory cytokine IL-10, pro-inflammatory chemokines CCL-3 and CXCL-1, and IL-6, a multifunctional cytokine with mainly systemic effects. To complement our analysis, we additionally supplemented *S100A9* ^-/-^ BMDMs with rS100A8. We reasoned that this would allow us to assess a direct function of S100A8/A9 during the cytokine response regulation upon *C. albicans* infection. For this purpose, mouse S100A8 was expressed in *E. coli*, purified (**Figure S4**), and verified by mass spectrometry. As previously reported (15, 40) S100A8 homodimers are used as functionally active substitute for the heterodimer which becomes inactive under cell culture conditions. Notably, rS100A8 was virtually LPS-free (see also *Materials and Methods* section). After 24 h *in vitro* infection, we quantified cytokine secretion levels of BMDMs from both genotypes using a multiplex ELISA approach (**Figures 2E–I**). Infected WT BMDMs were additionally treated with 930 µg/ml Paq and *S100A9* ^-/-^ BMDMs were supplemented with 10 µg/ml rS100A8. TNF, CCL-3, and IL-10 levels released by infected WT BMDMs were significantly reduced upon addition of Paq (**Figures 2E–G**). In agreement, TNF and CCL-3 release of infected *S100A9^-/-^* BMDMs was decreased compared to WT BMDMs, but increased considerably upon supplementation with rS100A8 (**Figures 2E, F**). In contrast, IL-6 and CXCL-1 release of infected WT BMDMs (**Figures 2H**, **3I**) was not affected by Paq, indicating that pharmacological inhibition under these conditions might not be as effective as genetic deletion of *S100A9*. Levels of IL-10 and IL-6 secretion by infected *S100A9^-/-^* BMDMs were similar to those of WT BMDMs. However, addition of rS100A8 induced exceeding levels of IL-10 (**Figure 2H**) and IL-6 (**Figure 2I**) compared to infected WT BMDMs, resulting in 10-fold and 100-fold higher secretion, respectively. This confirmed the boosting effect of S100A8/A9 on pro-inflammatory signaling pathways (17). In contrast, CXCL-1 levels declined upon treatment with rS100A8, whereas released levels from infected, but untreated WT and *S100A9^-/-^* BMDMs were similar (**Figure 2I**). Possibly, BMDMs require presence of intrinsic S100A8/A9 to release this chemokine in response to *C. albicans*. Hence, it seems plausible that administration of external rS100A8 could not reverse the effect either. In conclusion, Paq reduced the release of pro-inflammatory cytokines by infected BMDMs reflecting the genetic deletion of *S100A9* at least to some extent. This suggests that Paq treatment might also diminish hazardous effects of ICTD during experimental IAC.

### *In Vivo* Cytokine Analysis During Disseminated Peritonitis Reveals That Paq Treatment Reduces Levels of Pro-Inflammatory Cytokines and Chemokines

To assess the efficacy of Paq as possible treatment against ICTD during experimental IAC, we next investigated the effect of the compound on *in vivo* cytokine profiles. For this purpose, we IP-infected WT and *S100A9* ^-/-^ mice with *C. albicans* and harvested the livers after 24 h (**Figure 3**, WT + *Ca* and *S100A9* ^-/-^ + *Ca*). An additional group of WT mice (WT + *Ca* + Paq) was treated with Paq (30 mg/kg). The concentration was extrapolated from the BMDM experiments presented above (**Figure 2**) and adjusted to previous applications of the compound in mice (18). Furthermore, a group of *S100A9* ^-/-^ mice (*S100A9* ^-/-^ + *Ca* + rS100A8) was treated with rS100A8 (5 mg/kg). The concentration of rS100A8 was also extrapolated from BMDM experiments presented above (**Figure 2**). We chose the liver for the analysis, since IAC-induced ICTD was most prominent in this organ (**Figures 1B–D** **and** **Figure S2B**). We analyzed concentrations of 10 pro-inflammatory cytokine and chemokines, namely TNF, IL-1α, IL-1β, IL-12, CCL-3, CCL-4, GM-CSF, IL-10, IL-6, and CXCL-1 (**Figures 3A–J**).

Remarkably, the concentrations of 4 pro-inflammatory cytokines, namely TNF, IL-1α, IL-1β, IL-12 (**Figures 3A–D**) were significantly decreased in livers of Paq-treated WT and untreated *S100A9* ^-/-^ mice compared to untreated WT mice, whereas rS100A8 supplementation of *S100A9* ^-/-^ mice led to similar or even higher values than in WT mice. Moreover, secretion of the chemokines CCL-3 and CCL-4 was reduced by Paq treatment, but remained unaltered in livers of untreated *S100A9* ^-/-^ mice compared to those of untreated WT mice. Yet, the levels of both effectors were increased about 5-fold when *S100A9* ^-/-^ mice were supplemented with rS100A8 (**Figures 3E, F**). GM-CSF levels were not affected by Paq treatment of WT mice, however decreased in livers of knockout animals and the reduction was reverted by rS100A8 supplementation (**Figure 3G**). In contrast, levels of IL-10, IL-6, and CXCL-1 were either not affected or increased in livers of Paq-treated WT and untreated *S100A9* ^-/-^ mice, yet all three effectors were largely increased upon rS100A8 supplementation of *S100A9* ^-/-^ mice (**Figures 3H–J**).

In summary, we conclude that Paq has S100A8/A9-specific (TNF, IL-1α, IL-1β, IL-12) and pleiotropic anti-inflammatory effects (CCL-3 and -4) on cytokine responses during IAC which could be exploited for immune-targeted therapy.

### Paq Therapy Reduces ICTD Indu ced by Disseminated Peritonitis

**Figure 4:**
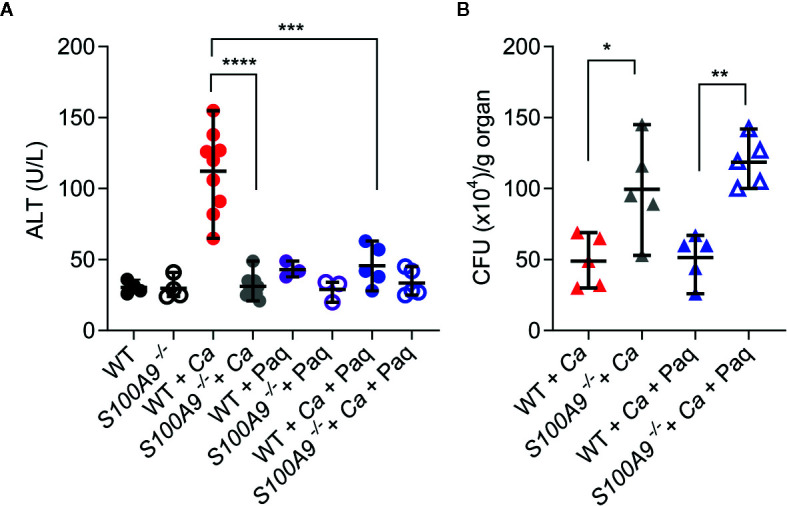
Paq reduces ICTD in experimental IAC without affecting fungal burden. WT and *S100A9* ^-/-^ mice were IP-infected with 3 × 10^6^ cells per g mouse. **(A)** Higher plasma ALT levels of infected WT mice (WT + *Ca*) during *C. albicans* IP infection compared to *S100A9* ^-/-^ mice (*S100A9* ^-/-^ + *Ca*), and during pharmacological inhibition of S100A9 (WT + *Ca* + Paq). **(B)** Inhibition of *S100A9* (WT + *Ca* + Paq) did not alter fungal burden when infected with *C. albicans* (WT + *Ca*), nor did Paq affect the increased fungal burden in mice lacking S100A9 (WT + *Ca* + Paq *vs*. *S100A9* ^-/-^ + *Ca* + Paq). **(A, B)** For ALT measurements, plasma (100 µl) from each sample was analyzed 24 h post infection using a Vetscan VS2 system. For fungal burden in livers, CFUs per g organ were determined 24 h post infection. Data is displayed as means ± SD and evaluated using an unpaired Mann-Whitney test. Each symbol represents an animal **(A)** WT (n = 4), *S100A9* ^-/-^ (n = 4), WT + *Ca* (n = 9) and *S100A9* ^-/-^ + *Ca* (n = 10), WT + *Ca* + Paq (n = 5), *S100A9* ^-/-^ + *Ca* + Paq (n = 5), WT + Paq (n = 3), *S100A9* ^-/-^ + Paq (n = 3). **(B)**: WT + *Ca* (n = 5) and *S100A9* ^-/-^ *+*(n = 5), WT + *Ca* + Paq (n = 5), *S100A9* ^-/-^ + *Ca* + Paq (n = 5)]. Statistical significance with * equals p-value < 0.05, **p-value < 0.01 ***p-value < 0.001, ****p-value < 0.0001.

Since Paq reduced pro-inflammatory cytokine release in mouse livers during experimental IAC, we aimed to test how Paq treatment of WT mice affects ICTD. Hence, we IP-infected two groups of WT mice with *C. albicans*, while one group was treated with Paq (30 mg/kg mouse) and the other group was mock-treated with PBS. Paq administration led to elimination of ICTD as indicated by reduced plasma ALT levels in infected mice to levels of uninfected animals (WT + *Ca* vs WT + *Ca* + Paq, **Figure 4A**) and similarly by reduced ocular discharge (compare **Figures S1A, S5A**). As expected, Paq treatment did not affect ALT levels in infected *S100A9* ^-/-^ mice (*S100A9* ^-/-^ + *Ca vs S100A9* ^-/-^ + *Ca* + Paq, **Figure 4A**). Furthermore, during disseminated IAC, S100A8/A9 deficiency led to increased fungal burden in the liver of infected animals (**Figure S2C**). To elucidate whether administration of Paq after 24 h could also affect fungal burden in infected WT mice, we harvested livers of infected WT and *S100A9* ^-/-^ mice, which were either Paq- or mock-treated. Paq treatment had no effect on the fungal burden in both mouse strains (*S100A9* ^-/-^ + *Ca vs S100A9* ^-/-^ + *Ca* + Paq, **Figure 4B**) indicating that potentially more pleiotropic anti-inflammatory effects of the compound did not affect fungal burden during experimental IAC.

Of note, these experiments confirm that despite fungal burden in livers of *S100A9* ^-/-^ mice was significantly higher than in WT mice, ICTD remained on levels of uninfected animals (see also **Figures S1A, S2B**). This suggests that ICTD during experimental IAC depends on the pro-inflammatory activity of S100A8/A9.

### S100A8/A9 Is Required for ICTD During Disseminated Peritonitis

The role of S100A8/A9 as alarmin during experimental IAC is currently unknown. To test the usefulness of recombinant protein therapy, we determined the effects of rS100A8 on the described mouse model of IAC. Since mouse rS100A9 forms homodimers with low activity, supplementation with rS100A9 did not lead to a reduction of ICTD in *C. albicans* peritonitis (*S100A9* ^-/-^ + *Ca vs. S100A9* ^-/-^ + *Ca* + rS100A9, **Figure S5B**). Therefore, as for the *in vivo* cytokine profiling (**Figure 3**) we administered purified rS100A8 protein (5 mg/kg mouse) to

**Figure 5:**
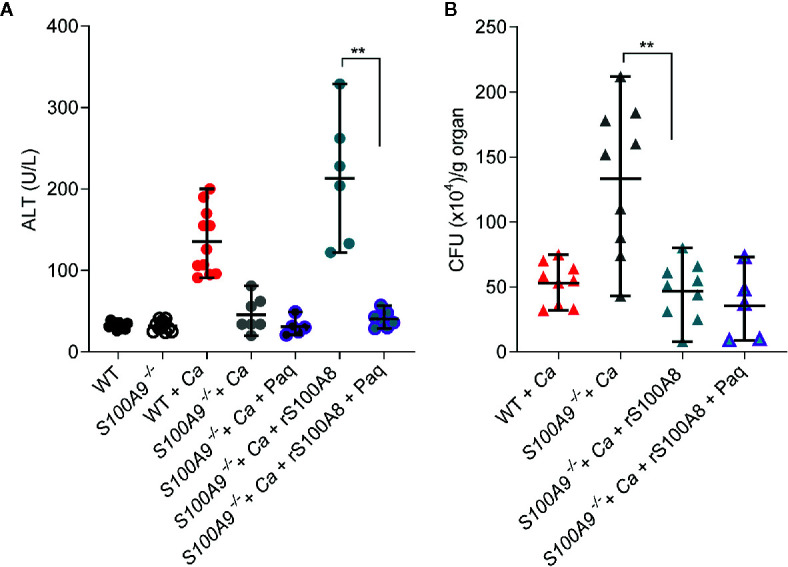
Recombinant S100A8 and Paq modify ICTD in experimental IAC. WT and *S100A9* ^-/-^ mice were IP-infected with 3 × 10^6^ cells per g mouse. **(A)** Restored basal levels of ALTs in rS100A8 complemented *S100A9* ^-/-^ mice that were treated with Paq (*S100A9* ^-/-^ + *Ca* + rS100A8 + Paq) indicate that Paq significantly reduced liver tissue damage during (*C.*) *albicans* infection compared to mice lacking Paq treatment (*S100A9* ^-/-^ + *Ca* + rS100A8). **(B)** rS100A8 injection into *S100A9* ^-/-^ mice (*S100A9* ^-/-^ + *Ca* + rS100A8) decreased fungal burden (*S100A9* ^-/-^ + *Ca* + rS100A8), but Paq treatment (*S100A9* ^-/-^ + *Ca* + rS100A8 + Paq) did not affect fungal clearance. **(A, B)** For ALT measurements, plasma (100 µl) from each sample was analyzed 24 h post infection using a Vetscan VS2 system. For fungal burden in livers, CFUs per g organ were determined 24 h post infection. Data is displayed as means ± SD and evaluated using an unpaired Mann-Whitney test. Each symbol represents an animal **(A)** WT (n =10), *S100A9* ^-/-^ (n = 10), WT + *Ca* (n = 11) and *S100A9* ^-/-^ + *Ca* (n = 7), *S100A9* ^-/-^ + *Ca* + Paq (n = 5), *S100A9* ^-/-^ + *Ca* + rS100A8 (n = 6), *S100A9* ^-/-^ + *Ca* + rS100A8 + Paq (n = 7). **(B)** WT + *Ca* (n = 9) and *S100A9* ^-/-^ + *Ca* (n = 9), *S100A9* ^-/-^ + *Ca* + rS100A8 (n = 9), *S100A9* ^-/-^ + *Ca* + rS100A8 + Paq (n = 5)]. Shown is data from two independent experiments, statistical significance with ** equals p-value < 0.01.

*S100A9* ^-/-^ mice infected with *C. albicans*. This supplementation of infected *S100A9* ^-/-^ mice increased ICTD as indicated by approximately 6-fold higher ALT plasma levels compared to untreated *S100A9* ^-/-^ mice (*S100A9* ^-/-^ + *Ca vs S100A9* ^-/-^+ *Ca* + rS100A8, **Figure 5A**) and similarly by increased ocular discharge (compare **Figures S1A, S5A**). Moreover, ALT plasma levels of infected, rS100A8-treated knockout mice were not significantly different from infected, but untreated WT mice (*S100A9* ^-/-^+ *Ca* + rS100A8 *vs* WT + *Ca*) suggesting that active S100A8 homodimers mimic heterodimers to cause WT levels of liver damage. S100A8/A9 is an antimicrobial protein against *C. albicans* (25, 26). In this context, the fungal clearance defect observed in infected *S100A9* ^-/-^ mice was remedied, reducing fungal load after supplementation of knockout animals with rS100A8 (*S100A9* ^-/-^ + *Ca vs S100A9* ^-/-^ + *Ca* + rS100A8, **Figure 5B**). Interestingly, the effect of rS100A8 administration on ALT plasma levels in infected *S100A9* ^-/-^mice could be reverted by treatment with Paq (*S100A9^-/^* ^-^ + *Ca* + rS100A8 *vs*. *S100A9^-/^* ^-^ + *Ca* + rS100A8 + Paq, **Figure 5A**). Expectedly, Paq administration to rS100A8-treated *S100A9* ^-/-^ mice did not affect fungal burden in the liver (**Figure 5B**).

Taken together, the data demonstrates that S100A8/A9 has a direct effect on ICTD and that the protein complex may serve as promising target for immune-directed therapy against IAC.

### Paq Has a Moderate Effect on Survival in Disseminated Peritonitis

**Figure 6:**
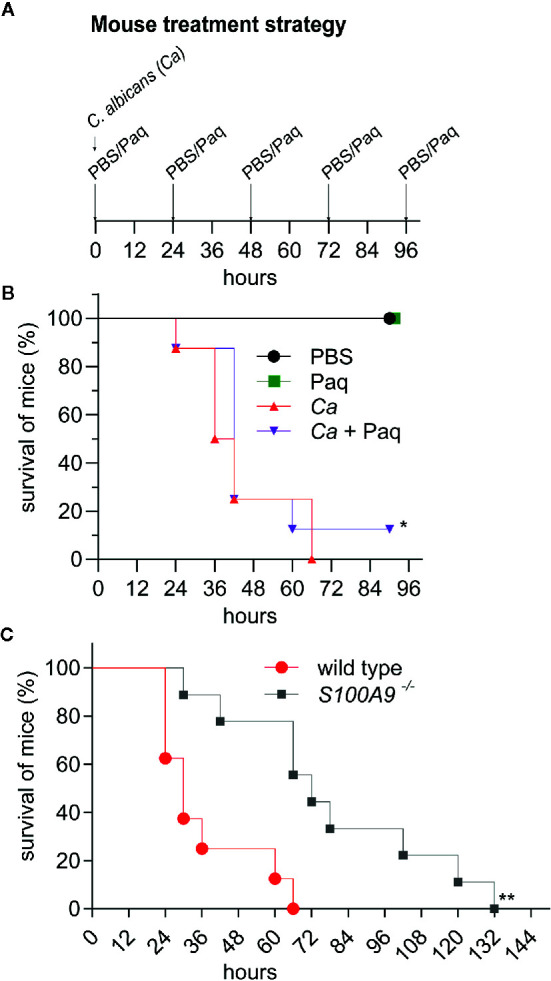
Paq-mediated inhibition of S100A8/A9 and lack of S100A8/A9 significantly delay host mortality during experimental IAC. IP infection with *C. albicans* (3 × 10^6^ cells per g mouse) in WT mice compared to Paq-treated WT mice and to *S100A9 ^-/-^* mice was performed and survival of animals was monitored. **(A)** Shown is the treatment strategy for **(B)**. WT mice were periodically treated with Paq (30 mg/kg) at 24-h intervals. **(B, C)** Displayed is the percentage of mouse survival over time. In **(B)** shown data indicates n = 2 mice per group (uninfected controls only treated with PBS or Paq were consistently healthy, see panel **(A)**, and **Figure 4**) and n = 8 infected mice per group (*Ca* and *Ca* + Paq) while in **(C)** shown data indicates n = 8 infected WT mice and n = 9 infected *S100A9^-/-^*mice. Statistical analysis for **(B, C)** was conducted on survival data using the log-rank test, statistical significance with * equals p-value < 0.05 and ** p-value < 0.01. Infected WT mice were considerably less active than *S100A9^-/-^*mice (see **Video S1**).

Next, we tested whether Paq administration could improve survival during experimental IAC. For this purpose, IP-infected WT mice were treated with daily doses of Paq (infection strategy, **Figure 6A**). Due to the severity of the experimental IAC model used, mice expectedly succumbed to infection within 72 h. Paq treatment resulted in a moderately increased survival rate five days post infection with a statistically significant effect (**Figure 6B**). Although the compound did not prevent all mice from succumbing to infection, Paq showed promising results, as between 24 and 48 h, treated mice survived longer compared to untreated mice (*Ca* + Paq *vs. Ca*, **Figure 6B**). Despite similar weight loss in infected mice observed between treated and untreated groups (**Figure S6A**), treated mice were observed to be more active during *C. albicans* infection. This was even more evident when WT and *S100A9* ^-/-^ mice were monitored for survival during *C. albicans* infection (**Figure 6C**). While IP-infected WT mice again succumbed to infection within 72 h, survival of *S100A9* ^-/-^ mice ranged at approximately 50% at this time point post infection (**Figure 6C**). Of interest were also the severe symptoms, such as for instance lethargy, found in WT mice between 18 and 30 h post infection, while *S100A9* ^-/-^ mice remained active during this time period (see **Video S1** for representative mice in **Figure 6B**). Nevertheless, similar to Paq treatment, mouse weight continued to decrease during infection in both WT and *S100A9* ^-/-^ mice (**Figure S6B**). Hence, our data suggest that using an anti-S100A8/A9 treatment as an adjuvant therapy could be a useful strategy to increase the therapeutic window for antifungal treatment during disseminated IAC.

## Discussion

This work characterizes the role of S100A8/A9 on host resolution of inflammation in an experimental model of disseminated *C. albicans* peritonitis. Most experimental models studying systemic fungal diseases use IV injection of fungal cells which bypass mucosal host defenses and establish an infection predominantly in the kidney and the brain (41). This study utilized an IAC model to induce systemic inflammation mimicking a severe clinical concern of postoperative *Candida* peritonitis (42).

Similar to IV-, IP-induced infection allows rapid blood dissemination of pathogens with exposure to an active population of phagocytes, complement cascade activation, and subsequent abscess formation in the peritoneal cavity (42). Rapid fungal dissemination during experimental IAC was evident from considerably high *C. albicans* CFUs in the liver, spleen, and kidney of infected animals 24 h after inoculation (**Figure 1A**). In intravenous infection, in contrast to the IP injection route, ALT plasma levels did not increase above uninfected control within the first 48 h of infection (**Figure S6C**) and hence the peritonitis infection model is suitable to describe the acute and severe onset of disseminated infection leading to ICTD in liver tissue (**Figure 1B** and **Figure S2B**).

To determine the duality of S100A8/A9 during disseminated IAC, we used *S100A9* ^-/-^ mice, which also lack the S100A8 protein subunit despite normal RNA levels, and thus the mice represent a functional S100A8/A9 double-knockout strain (30). On the one hand, the liver was among the most heavily infected organs during experimental IAC and fungal burden was consistently higher in *S100A9* ^-/-^ compared to WT mice (**Figures 1A**, **4B**, **5B** and **Figure S2C**). In other words, fungal clearance in WT livers was approximately 3-times more efficient when compared to *S100A9^-/-^* livers. These findings suggest that the presence of S100A8/A9 was required for fungal clearance during experimental IAC, and supports our previous notion that antimicrobial properties of S100A8/A9 are essential during *C. albicans* infection (26). On the other hand, the presence of S100A8/A9 in WT mice resulted in significantly more liver damage compared to *S100A9* ^-/-^ mice, as indicated by ALT plasma levels, despite higher fungal load (**Figures 1B**, **4A**, **5A**, and **Figure S2B**). This implies that the induction of S100A8/A9-mediated inflammatory responses considerably contributed to tissue damage. Interestingly, other clinical blood parameters did not indicate further organ damage, such as for instance kidney injury or renal dysfunction (**Figure 1B**). Therefore, we assumed that hazardous ICTD was most prominent in the liver during experimental IAC. The various leucocyte infiltration zones observed in WT liver tissues compared to those from *S100A9* ^-/-^ mice (**Figure 1C**) indicated that more frequent accumulations of leukocytes coincide with fungal clearance in WT mice. Also, the organ-wide granulocytic infiltrate was slightly lower in *S100A9* ^-/-^ than in WT mice, but not to a significant extent (**Figure S2A**). This might suggest that the degree of concentration and activation of granulocytes could be increased in WT livers, leading to more organ-wide inflammation, as also corroborated by comprehensive inflammatory scoring of histological liver sections (**Figure 1D**). These findings support the association of the S100 family of proteins with inflammatory disorders (17), and our data indicates that S100A8/A9 activity depends on the modulation of both antimicrobial and inflammatory activity.

To broaden our insight into S100A8/A9-mediated ICTD we performed cytokine analyses using BMDMs from WT and *S100A9* ^-/-^ mice which were infected with *C. albicans in vitro*. In accordance with plasma ALT levels and histological examinations from IP-infected mice, release of pro-inflammatory TNF was reduced in infected *S100A9^-/-^* BMDMs compared to WT cells (**Figure 2A**).

Based on these results our next aim was to test whether S100A8/A9-mediated effects on the macrophage cytokine response could be altered by mimicking the genetic deletion with a compound specifically blocking the PRR binding by S100A8/A9. One such compound is Paq, previously used to treat inflammatory disorders, such as lupus erythematosus (18, 21). Studies have shown that Paq binds to the S100A9 subunit of S100A8/A9, thereby selectively blocking the pro-inflammatory activity of the heterodimer (27). In addition to treating infected WT BMDMs with Paq, we complemented *S100A9^-/-^* BMDMs with rS100A8, to elucidate whether the observed effects were directly linked to the protein complex. Of note, the use of rS100A8 homodimer as substitute for the heterodimer is widely accepted (17), since S100A8/A9 swiftly tetramerizes and subsequently loses its pro-inflammatory activity under *in vivo* and cell culture conditions. Studies using experimental models of chronic inflammation indicated that S100A8/A9 activity must be regulated in such a timely fashion to protect from systemic inflammatory damage (17). Pro-inflammatory effects of rS100A8 are thus likely to be more effective and prolonged than those of S100A8/A9. In our *in vitro* macrophage infection experiments, Paq reduced release of TNF, pro-inflammatory chemokine CCL-3, and anti-inflammatory IL-10 from WT BMDMs infected with *C. albicans* to levels similarly low as from infected *S100A9* ^-/-^ BMDMs (**Figures 2E–G**). Accordingly, rS100A8 restored release of TNF, CCL-3, and IL-10 to higher levels than of WT BMDMs (**Figures 2E–G**), most probably due to the above-mentioned characteristics of rS100A8. While release of IL-6 was largely unaffected by genetic deletion of *S100A9* ^-/-^ and Paq treatment, rS100A8 restored release to higher levels than of WT BMDMs (**Figure 2H**). Release of neutrophil-attractant CXCL-1 from Paq-treated, infected WT cells was slightly higher than of untreated WT cells. However, *S100A9* ^-/-^ BMDMs were virtually unable to release CXCL-1 in response to *C. albicans* and secretion could neither be restored by addition of rS100A8 (**Figure 2I**). CXCL-1 release appears to be hampered in *S100A9* ^-/-^ BMDMs. Levels in uninfected cells are significantly lower than in WT BMDMs, only to decrease further upon *C. albicans* challenge. Hence, lack of S100A8/A9 expression might cause intrinsic blockage of CXCL-1 release by BMDMs probably by an unknown mechanism, which external rS100A8 is unable to revert.

To confirm whether Paq and rS100A8 could have similar effects on cytokine responses in the liver of mice during experimental IAC, we analyzed 10 cytokines and chemokines in the respective liver homogenates (**Figure 3**). In summary, the effects of Paq and rS100A8 on liver cytokine responses during experimental IAC could be categorized into four groups. 1) Reduced by pharmacological intervention and by genetic deletion: TNF, IL-1α, IL-1β, and IL-12; 2) Reduced by pharmacological intervention but not by genetic deletion: CCL-3 and CCL-4; 3) Not reduced by pharmacological intervention but by genetic deletion: GM-CSF; 4) Not reduced by pharmacological intervention and neither by genetic deletion, however increased by rS100A8 administration: IL-10, IL-6, and CXCL-1. The reduced levels of pro-inflammatory cytokines of group 1 could indeed contribute to alleviation of ICTD observed in Paq-treated and *S100A9* ^-/-^ mouse livers during experimental IAC. This notion is confirmed by results showing that Paq treatment also reduced plasma ALT to levels comparable with those of infected *S100A9* ^-/-^ mice or of uninfected controls (**Figure 4A**). Effects of Paq on reduced chemokine CCL-3 and -4 release, which were absent in *S100A9* ^-/-^ mice (group 2), could be explained by previously described pleiotropic anti-inflammatory activities of the compound (18). Cases in which genetic deletion of *S100A9* ^-/-^ reduced cytokine levels, but not treatment with Paq (group 3), could be caused by insufficient blockage of the entirety of available S100A8/A9 molecules or possibly by unknown receptor bindings which are unaffected by S100A9-Paq interaction. Cytokines and chemokines of group 4 were not significantly affected or increased upon Paq treatment and genetic deletion of S100A8/A9 in livers during experimental IAC. Possibly their regulation is compensated by other signaling mechanisms. The fact that injection of rS100A8 induced increased production of IL-10, IL-6, and CXCL-1 in liver of infected *S100A9* ^-/-^ mice, reiterates the possibly prolonged effect of rS100A8. In contrast to BMDMs, CXCL-1-producing cells in the liver, such as endothelial and epithelial cells, might not be affected in the same manner.

Of note, our data demonstrates that ICTD, which was alleviated in *S100A9* ^-/-^ mice, could be restored by injection of rS100A8 as indicated by plasma ALT levels (**Figure 5A**). Thus, at least to a large proportion the reported effect on ICTD in experimental IAC is dependent on S100A8/A9. Albeit administration of rS100A8 led to enhanced fungal clearance in the liver of infected *S100A9* ^-/-^ mice (**Figure 5B**), the activity of S100A8 has the potential to induce dire effects on host-mediated tissue damage probably disqualifying recombinant therapy with rS100A8 in humans. In contrast, selective pharmacological inhibition of pro-inflammatory activities of the protein complex could be beneficial in patients with IAC. Surprisingly, we found that Paq also reverted ICTD in infected *S100A9* ^-/-^ mice previously treated with rS100A8 (**Figure 5A**). Bjork *et al*., showed strong binding of Paq to human and mouse S100A9 and negligible binding to human S100A8 *in vitro*; however, binding to mouse S100A8 was not investigated (27). Notably, it may also be possible that Paq exerts above-mentioned other immune-modulatory effects that give rise to the reduction seen in **Figure 5A**.

Paq did not cause cell toxicity in BMDMs (**Figure S3**) and no adverse effects in mice at the concentrations used for animal experiments (**Figure 6B**). Hence, Paq possibly represents a promising adjuvant therapeutic option to dampen hazardous ICTD and to enlarge the therapeutic window for targeting the pathogen with antifungal drugs (**Figure 6B**). This is particularly relevant, since the compound had no adverse effect on fungal clearance. The results additionally support the notion of an uncoupled nature of S100A8/A9’s pro-inflammatory and antimicrobial activities which depend on binding of different ions to distinct binding sites on the protein complex. Furthermore, *S100A9^-/-^* mice showed increased survival compared to WT mice (**Figure 6C**), although antimicrobial activity of S100A8/A9 is also required for fungal clearance (**Figure 5B**). This suggests that at early time points during experimental IAC the damage inflicted by ICTD is more hazardous than the extent of fungal burden *per se*. To further develop such a host-targeted therapeutic approach, an improved treatment regimen and probably more stringent inhibitory molecules are necessary.

**Figure 7:**
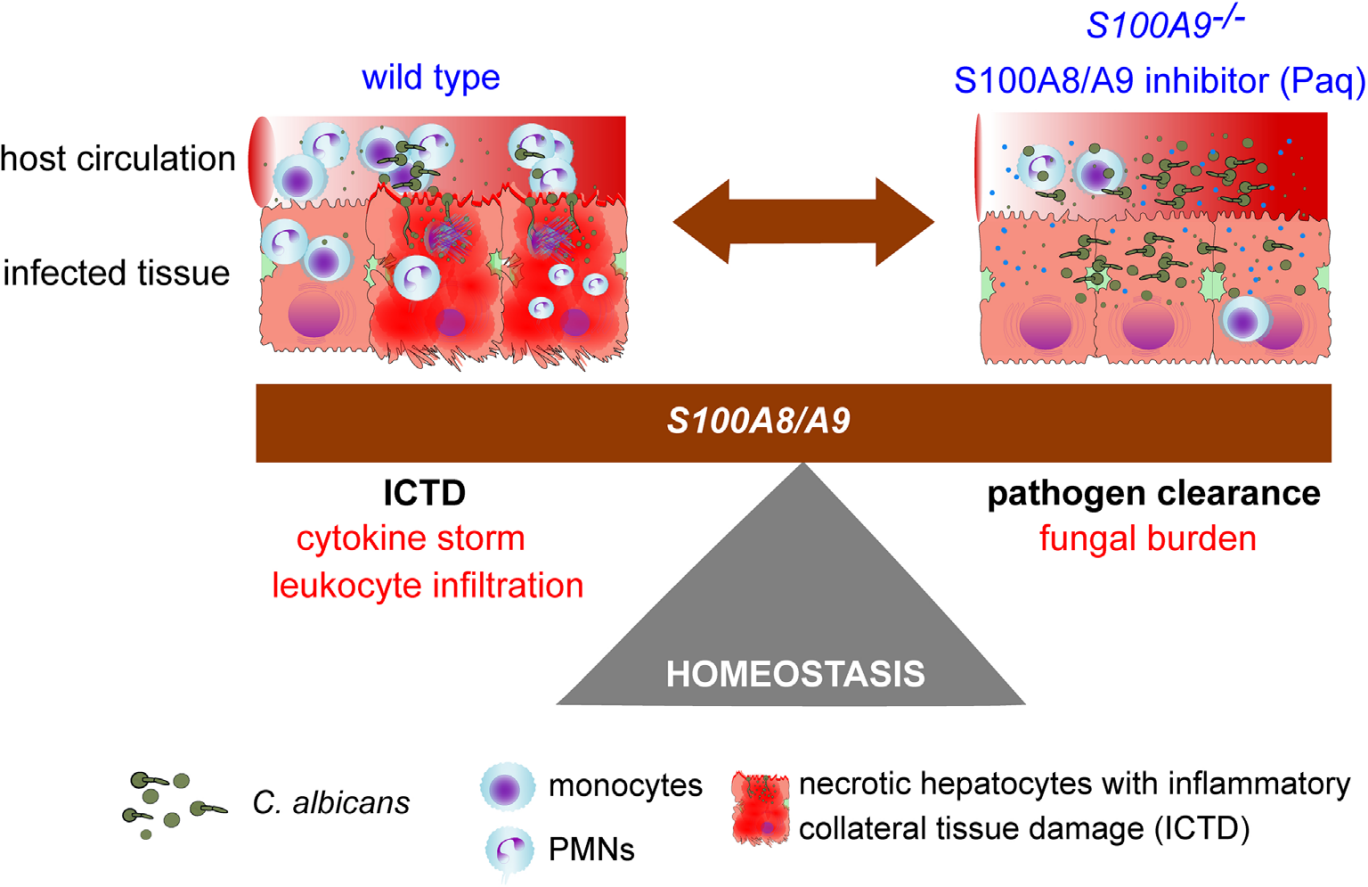
Study summary showing the homeostasis dilemma in the modulation of S100A8/A9. The host requires S100A8/A9 for fungal clearance (*S100A9^-/-^)*, but during systemic peritonitis (wild type), the heterodimer induces pro-inflammatory factors that may lead to organ failure driven by ICTD.

Fungal-host interactions induce local innate immune cells to release pro-inflammatory cytokines that further increase immune cell infiltration and inflammation in the effort of fungal clearance, but at the cost of elevated tissue damage. This study underlines the dilemma between pathogen clearance and ICTD, or in other words the protective and harmful roles of S100A8/A9 during experimental IAC (**Figure 7**). With S100A8/A9 at site, immune homeostasis needs to be tightly controlled to reduce ICTD induced by a multitude of cytokines and immune effector cells, potentially causing organ failure and death. But without S100A8/A9 at site, pathogens might not be sufficiently eradicated to be kept under control, potentially causing life-threatening infections. Currently, there are no specific immune-targeted therapies against severe microbial infections, such as sepsis, and management often focuses on containing the infection through source control, antimicrobial therapy, and organ function support (43). Here we present a potential adjuvant therapy that may support the patient’s resilience upon life-threatening infection and thereby prolong the window of opportunity for proper fungal diagnostics and targeted therapy. Considering this, future adjuvant therapeutics, such as Paq, could be an essential element of reducing morbidity and mortality of life-threatening fungal infections.

## Data Availability Statement

The raw data supporting the conclusions of this article will be made available by the authors, without undue reservation.

## Ethics Statement

Animal experiments and isolation of primary cells were carried out following the recommendations in the Guide for the Care and Use of Laboratory Animals, conformed to Swedish animal protection laws and applicable guidelines (djurskyddslagen 1988:534; djurskyddsförordningen 1988:539; djurskyddsmyndigheten DFS 2004:4) in a protocol approved by the local Ethical Committee (Umeå djurförsöksetiska nämnd, permit numbers A12-13, A80-14, A79-14, A21-2020). Written informed consent was obtained from the owners for the participation of their animals in this study.

## Author Contributions

CU provided funding and together with MS and NU designed the study. TV and JR contributed to the conceptualization of the study. MS, NU, and EB collected and analyzed the data. Manuscript drafting was performed by NU, MS, and CU. SH contributed with preparation of histological sections. MN provided data from intravenous infection and supported manuscript editing. All authors contributed to the article and approved the submitted version.

## Funding

We are grateful for funding provided to CU by the Swedish research council VR-M 2014-02281 and VR-M 2017-01681, the Kempe Foundation SMK-1453, the Åke Wiberg Foundation M14-0076 and M15-0108, and the Medical Faculty of Umeå University 316-886-10. NU and MS held a postdoctoral fellowship received in competition from UCMR. JR and TV were funded by grants of the Interdisciplinary Center of Clinical Research at the University of Münster (Ro2/023/19 and Vo2/011/19) and the German Research Foundation CRC 1009 B08 (JR) and B09 (TV). The funders had no rule in study design nor analysis and interpretation of the results

## Conflict of Interest

The authors declare that the research was conducted in the absence of any commercial or financial relationships that could be construed as a potential conflict of interest.

## Supplementary Material

Supplementary Video 1 S100A8/A9 deficiency delays peritonitis symptoms that manifest during intraperitoneal candidiasis with WT mice being virtually immobile. WT and *S100A9^–/-^* IP-infected mice with 3 × 10^6^ C*. albicans* per g mouse are shown. At time point 18 h, WT mice were inactive, hunched, and 63% of mice succumbed to infection within 30 h (see **Figure 6C**, WT). In contrast, *S100A9^-/-^* mice were more active, and 12% of mice succumbed to infection after 30 h (**Figure 6C**, *S100A9^-/-^*).
