# Supplementary figures and images for: Immune Resolution Dilemma: Host Antimicrobial Factor S100A8/A9 Modulates Inflammatory Collateral Tissue Damage During Disseminated Fungal Peritonitis
Source: Front Immunol. 2021 Feb 26;12:553911. doi: 10.3389/fimmu.2021.553911 (PMC7953150; doi:10.3389/fimmu.2021.553911)

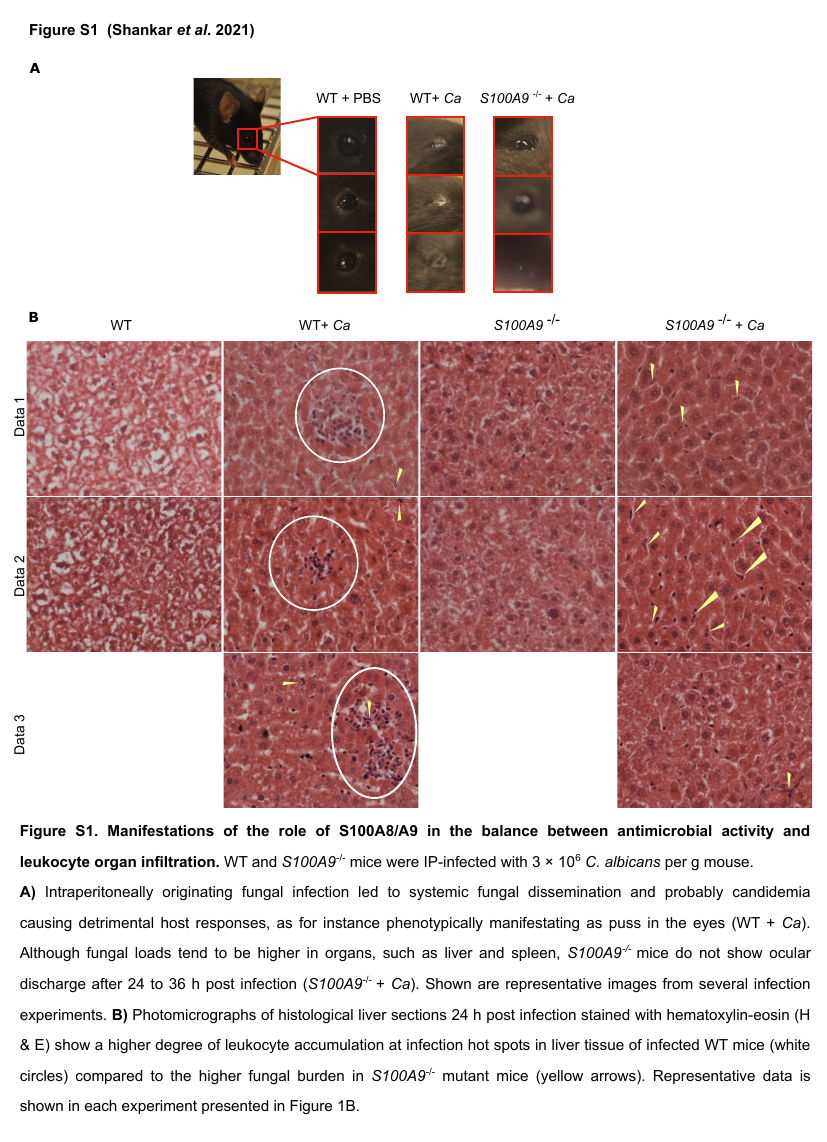

Supplement: Supplementary file 2 [file Image_1.tif]

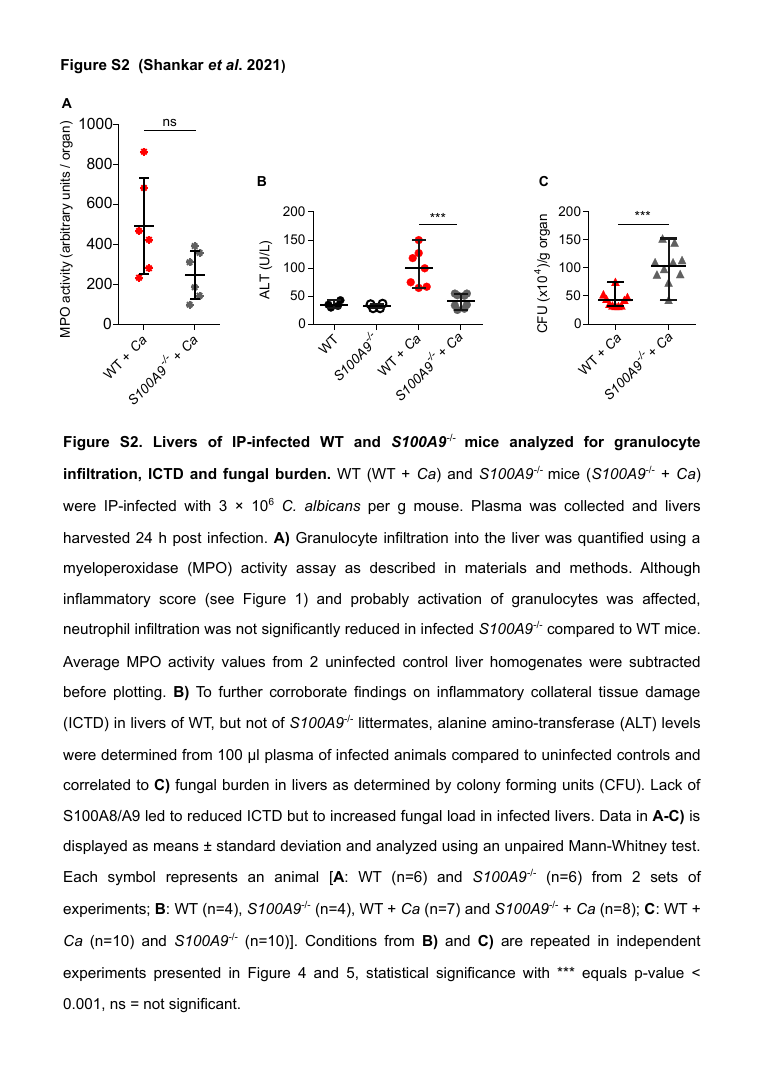

Supplement: Supplementary file 3 [file Image_2.tif]

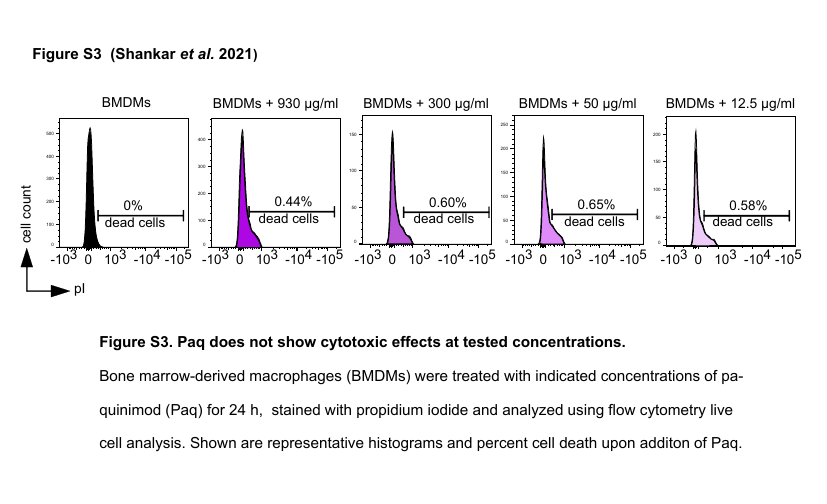

Supplement: Supplementary file 4 [file Image_3.tif]

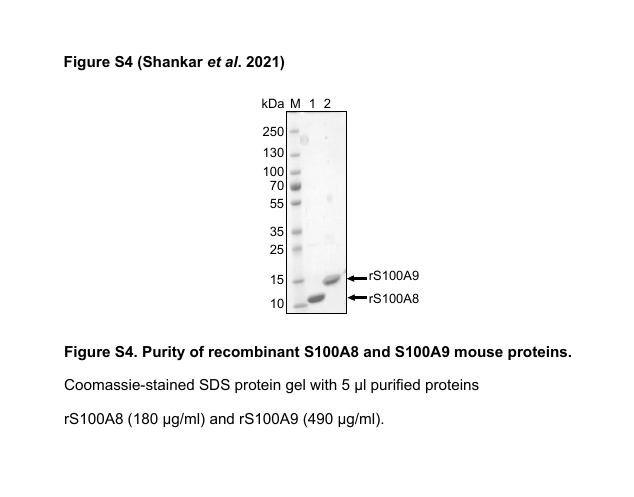

Supplement: Supplementary file 5 [file Image_4.tif]

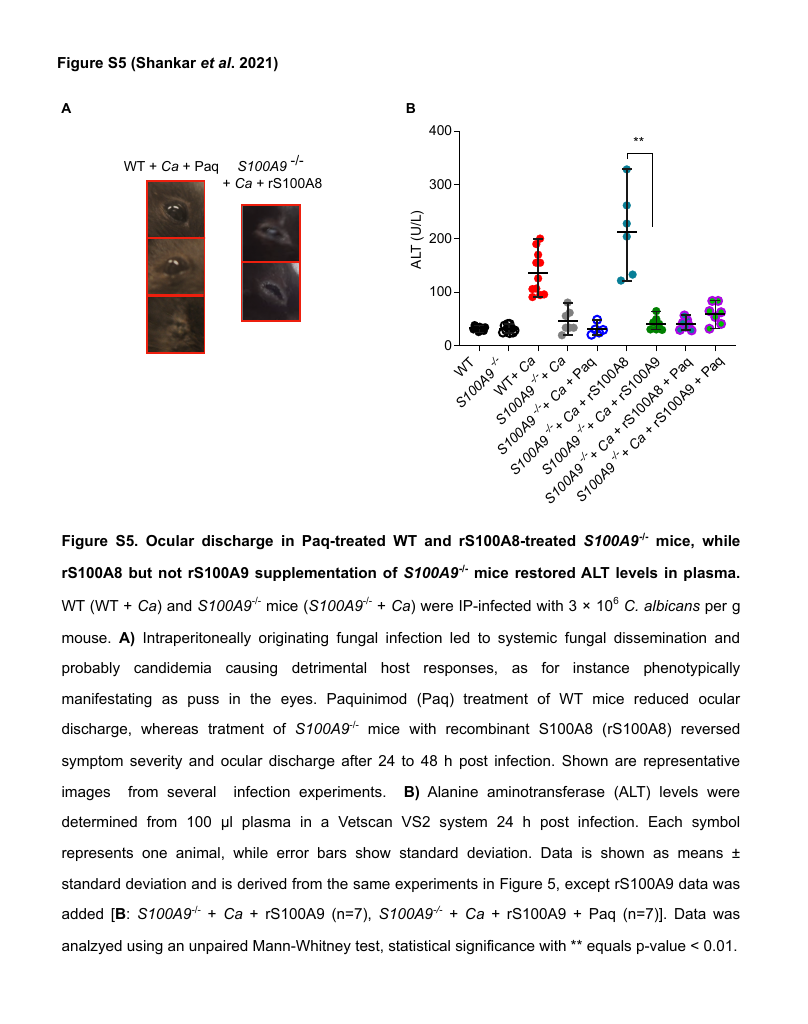

Supplement: Supplementary file 6 [file Image_5.tif]

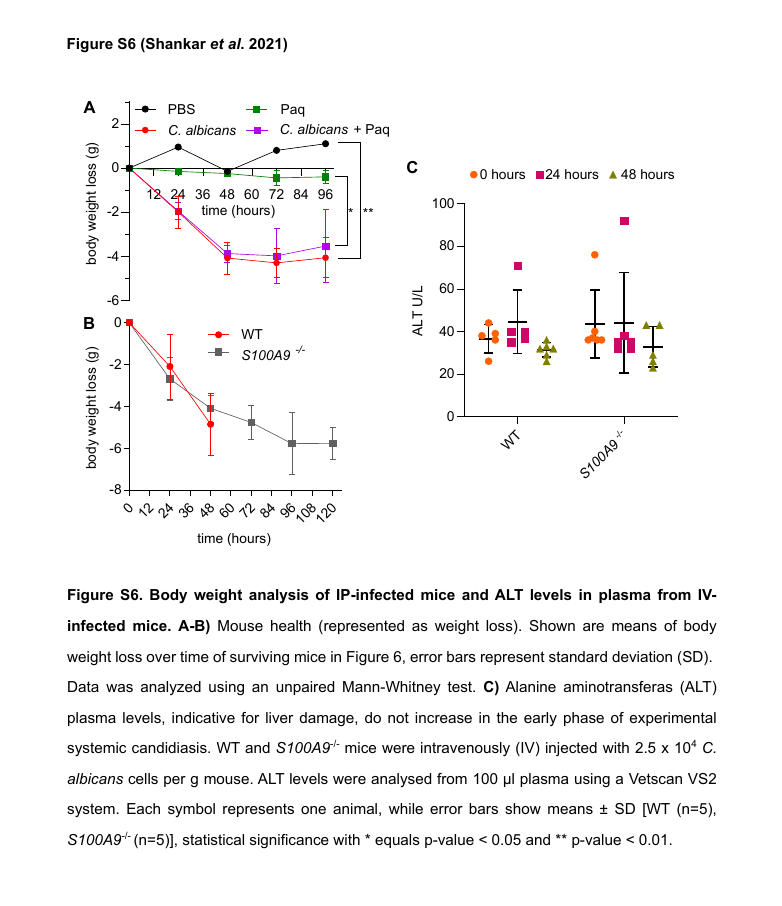

Supplement: Supplementary file 7 [file Image_6.tif]
